# Fungal Laccases: Production, Function, and Applications in Food Processing

**DOI:** 10.4061/2010/149748

**Published:** 2010-09-21

**Authors:** Khushal Brijwani, Anne Rigdon, Praveen V. Vadlani

**Affiliations:** Bioprocessing Laboratory, Department of Grain Science and Industry, Kansas State University, Manhattan, KS 66506, USA

## Abstract

Laccases are increasingly being used in food industry for production of cost-effective and healthy foods. To sustain this trend widespread availability of laccase and efficient production systems have to be developed. The present paper delineate the recent developments that have taken place in understanding the role of laccase action, efforts in overexpression of laccase in heterologous systems, and various cultivation techniques that have been developed to efficiently produce laccase at the industrial scale. The role of laccase in different food industries, particularly the recent developments in laccase application for food processing, is discussed.

## 1. Introduction

Laccase (benzenediol: oxygen oxidoreductase, EC 1.10.3.2) is a part of broad group of enzymes called polyphenol oxidases containing copper atoms in the catalytic center and are usually called multicopper oxidases. Laccases contain three types of copper atoms, one of which is responsible for their characteristic blue color. The enzymes lacking a blue copper atom are called yellow or white laccases. Typically laccase-mediated catalysis occurs with reduction of oxygen to water accompanied by the oxidation of substrate. Laccases are thus oxidases that oxidize polyphenols, methoxy-substituted phenols, aromatic diamines, and a range of other compounds [1]. 

Laccases are widely distributed in higher plants and fungi [2] and have also been found in insects and bacteria [3]. Laccases are distributed in Ascomycetes, Deuteromycetes, and Basidiomycetes, being particularly abundant in many white rot fungi that are involved in lignin metabolism [4, 5]. Owing to the higher redox potential (+800 mV) of fungal laccases compared to plants or bacterial laccases they are implicated in several biotechnological applications especially in the degradation of lignin [6]. For instance, redox potentials of laccases from common laccase producing fungi are reported as 790 mV (*Trametes villosa*), 450 mV (*Myceliophthora thermophila*), 750 mV (*Pycnoporus cinnabarinus)*, and 780 mV (*Botrytis cinerea*) [7]. Laccases, therefore, possess excellent potential to be used as processing aids for the food industry. 

The successful application of laccases in food processing would require production of high amounts at reduced costs. Several production strategies can be adopted along with media and process optimization to achieve better process economics. Concomitantly, overexpression of laccase in suitable host organisms would provide means to achieve high titers. Use of inducers could also enhance production capabilities [8]. 

The main objective of this paper is to summarize the wealth of information available in the literature with regard to laccase mechanism, production, and overexpression and eventually its role in food processing. Laccase has several food-based applications including bioremediation, beverage (fruit juice, wine and beer) stabilization, uses in baking industry, and role in improvement of overall food quality. The versatility of laccase in its action and its wide occurrence in several species of fungi contribute to the easy applicability in biotechnological processes. The present review, therefore, should help to shed light on the general characteristics of laccase in an effort to create a database that could aid usage of laccase in the food processing industry.

## 2. Mechanism of Laccase Action

The catalysis of laccase occurs with reduction of one molecule of oxygen to water accompanied with one electron oxidation of a wide range of aromatic compounds which includes polyphenols [9], methoxy-substituted monophenols, and aromatic amines [4]. This oxidation results in generation of oxygen-centered free radical that can be converted to quinone in a second enzyme catalyzed reaction. Laccase catalysis occurs in three steps: (1) type I Cu reduction by substrate; (2) electron transfer from type I Cu to the type II Cu and type III Cu trinuclear cluster; (3) reduction of oxygen to water at the trinuclear cluster [10].

**Figure 1:**
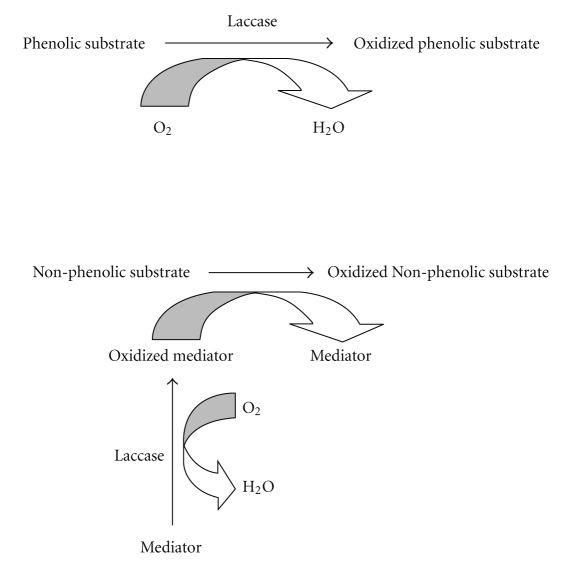
Mechanism of laccase action for both phenolic and nonphenolic substrates.

The laccase mediated catalysis can be extended to nonphenolic substrates by the inclusion of mediators. Mediators are a group of low molecular weight organic compounds that can be oxidized by laccase first forming highly active cation radicals capable of oxidizing nonphenolic compounds that laccase alone cannot oxidize (Figure 1). The most common synthetic mediators are 1-hydro-xybenzotriazole (HOBT), N-hydro-xyphthalimide (NHPI), and 2,2′-azinobis-3-ethylthiazoline-6-sulfonat (ABTS) [11].

## 3. Occurrence of Laccase in Fungal Systems

Laccase activity has been demonstrated in several fungal species leading to the notion that most of all fungi produce laccase. This, however, should not be generalized as there are several physiological groups of fungi that apparently do not produce laccase. Laccase production has never been demonstrated in lower fungi, that is, *Zygomycetes* and *Chytridiomycetes* [12]. Several reports can be referred, in the literature on production of laccase in ascomycetes such as *Gaeumannomyces graminis* [13], *Magnaporthe grisea* [14], and *Ophiostoma novo-ulmi *[15], *Mauginella* [16], *Melanocarpus albomyces* [17], *Monocillium indicum *[18], *Neurospora crassa* [19], and *Podospora anserina *[20]. In addition to plant pathogenic species, laccase production was also reported for some soil ascomycete species from the genera *Aspergillus*,* Curvularia* and *Penicillium* [21–23], and in some freshwater ascomycetes [24, 25]. 

Wood degrading ascomycetes like *Trichoderma *and *Botryosphaeria *have been shown to have some laccase activity. While *Botryosphaeria* produces constitutively a dimethoxyphenol oxidizing enzyme that is probably true laccase [26] there are only some strains of *Trichoderma* that exhibit low level production of a syringaldazine oxidizing enzyme [27]. In case of wood rotting xylariaceous ascomycetes, two strains of *Xylaria sp.* and one of *Xylaria hypoxylon* exhibited syringaldazine oxidation [28]. In complex liquid media, the fungi *X. hypoxylon* and *Xylaria polymorpha* produced appreciable titers of an ABTS oxidizing enzyme [29]. Furthermore, ascomycete species closely related to wood-degrading fungi which participate in the decay of dead plant biomass in salt marshes have been shown to contain laccase genes and to oxidize syringaldazine [30]. Basidiomycete yeast like *Cryptococcus neoformans* produces a true laccase capable of oxidation of phenols and aminophenols and is unable to oxidize tyrosine [31]. The production of laccase was not demonstrated in ascomycetous yeasts, but the plasma membrane bound multicopper oxidase Fet3p from *Saccharomyces cerevisiae* shows both sequence and structural homology with fungal laccase [32, 33]. 

Wood rotting basidiomycetes causing white rot and a related group of litter decomposing saprotrophic fungi are the most widely known species that produce appreciable quantity of laccase. Almost all species of white rot fungi were reported to produce laccase to varying degree [34]. In case of *Pycnoporus cinnabarinus* laccase was described as the only ligninolytic enzyme produced by this species that was capable of lignin degradation [35]. Brown-rot fungi on the other hand are not known, in general, to carry laccase production capabilities. A DNA sequence with relatively high similarity to that of laccase was detected in *Gloeophyllum trabeum* that was capable of oxidizing ABTS [36]. Though no laccase protein has been purified from brown-rot species, the oxidation of syringaldazine has recently been detected in the brown-rot fungus *Coniophora puteana *[37] and the oxidation of ABTS was reported in *Laetiporus sulphureus* [38]. 

## 4. Overexpression of Laccase

Due to the ability of fungal laccases to oxidize phenolic and nonphenolic aromatic compounds, increased interest in the application of these enzymes for various industrial applications, including food, pulping, textile, wastewater treatment, and bioremediation, is growing greatly [8]. To successfully utilize laccases in these applications, production of large quantities at a low cost is essential. 

To make laccases available for industrial applications, methods to reduce costs include fermentation media optimization, novel fermentation methods, and genetic modification for large scale production via eukaryotic recombinant strains. Much research has been done to identify effective methods for mass production of laccase using the above mentioned methods. Determination of optimum fermentation media can easily be achieved but cofactors and inducer compounds can cause an undesirable increase in cost during growth at industrial scale. Novel fermentation methods can also cause undesirable increases to cost due to modifications to preexisting facilities. Genetic modification presents a promising method of overexpression of laccase for large applications. However, fungal laccases require posttranslational modifications (glycosylation), which only eukaryotic microorganisms are capable of carrying out creating limitations for genetic manipulation for overexpression of laccase. Laccase genes have been successfully cloned and heterologously expressed in the filamentous fungi *Aspergillus niger*, *Aspergillus oryzae*, and *Trichoderma reesei* [8]. Only a few bacterial laccases have been thoroughly studied to reveal industrial advantages over fungal laccases. Bacterial laccases have been found to be highly active and have higher stability at higher temperatures and pH values compared to fungal laccases [39]. Laccase-like enzymes isolated from bacterial cultures have been found to be very similar to fungal laccases; however, they vary in activity [39].

Research by [40] focused on optimizing media conditions using multiple micronutrients for maximum production of laccase by a previously identified fungal strain belonging to the genus *Gandoderma* and referred to as *WR-1*. Strain *WR-1*, a white-rot fungus, was isolated from tree bark using tissue culture techniques and was found to produce high amounts of laccase during fermentation. *WR-1* was naturally found to show laccase activity of 124 U/ml, compared to typical strains which show activity in a range of 4–100 U/ml. The experimental design for determining the optimum media conditions included the use of the orthogonal matrix method. This allowed for the statistical evaluation of the relative importance of various nutrients for the highest production of laccase using submerged fermentation methods. It was concluded that *WR-1* produced increasing amounts of laccase when grown in a starch-based medium with the addition of copper sulphate and 2, 5-xylidine, as a laccase production inducer. *WR-1* was able to increase laccase production to 692 U/ml during fermentation in the optimized media, a significant increase compared to other strains under similar fermentation conditions [40].

 To help increase laccase production, research has focused on using recombinant fungal strains for maximum production. Research by [41] successfully transferred laccase genes from the basidiomycete *Tramete hirsuta* into the ascomycete *Penicillium canescens* for heterologous expression. The fungal strain from the genus *Penicillium* was chosen due to its ability to secrete large amounts of enzyme into culture media and it has been demonstrated that synthesized enzymes are safe for human consumption. After successful transformation, it was found that 98% of the target enzyme activity was detectable in the liquid culture medium. It was also found that the molecular weight of the recombinant enzyme matched the native laccase produce by *T. hirsuta* [41]. Further research is still needed to ensure high laccase production using large-scale fermentation methods.

Additionally, research by [53] focused on transforming laccase genes from *Trametes versicolor* into the methyltrophic yeast *Pichia pastoris* for heterologous expression. The *P. pastoris *expression system is commonly used to achieve high expression levels of heterologous proteins. This yeast has been found to achieve high cell densities during growth in a minimal media in a short period of time. Furthermore, *P. pastoris* has been found to be an efficient secretion system and capable of posttranslational modifications (e.g., glycosylation). After successful transformation of the *P. pastoris* expression system, it was found that utilizing a solid-state fermentation (SSF) method produced similar laccase production results compared to submerged fermentation (SmF) methods [53]. 

Recently, a few bacterial laccases have been isolated from *Escherichia coli*, *Bacillus halodurans*, *Thermus thermophilus,* and several species of *Streptomycetes*. Little is known about the function of laccases in bacterial physiology but they are believed to play a role in melanin production, spore coat resistance, morphogenesis, and detoxification of copper [54]. The bacterial laccase CotA isolated from *Bacillus subtilis* was found to be an endospore coat protein with high thermostability [55]. Utilizing bacterial laccases for industrial production would allow for new biotechnological applications due to the ease of genetic improvements to expression level, activity, and selectivity [56]. The combination of random and site-directed mutagenesis was used to produce a double mutant with improved functional expression in *E. coli* and improved specific activity for different dyes [56]. Wu et al. [39] isolated a new strain of *Aeromonas hydrophilia* designated WL-11 from activated sludge in an effluent treatment plant of a textile and dyeing industry. The gene encoding laccase was cloned from the newly isolated strain and successfully expressed in *E. coli* BL21(DE3). The recombinant strain produced a high level of laccase compared to the wild type. The recombinant laccase was characterized and could be used as a biocatalyst in biotechnological applications requiring large quantities of laccase [39].

## 5. Production Systems for Laccase

Laccases are extracellular enzymes secreted into the medium by filamentous fungi [57]. Laccases are generally produced during the secondary metabolism of different fungi. Several factors including type of cultivation (submerged or solid state), carbon limitation, nitrogen source, and concentration of microelements can influence laccase production [58]. Subsequent sections delineate the role of different process parameters in laccase production.

## 6. Influence of Carbon and Nitrogen Source on Laccase Production

The excessive concentrations of glucose are inhibitory to laccase production in various fungal strains [37]. An excess of sucrose also reduced the production of laccase by blocking its induction and only allowed constitutive production of enzyme. Use of polymeric substrates like cellulose was able to alleviate this problem [37]. Fungal laccases are often triggered by nitrogen depletion [59], but it was also found that in some strains nitrogen had no effect on enzyme activity [60]. High laccase activity was reported in some studies using low carbon to nitrogen ratio [61], but other studies showed that higher laccase production was achieved at high carbon to nitrogen ratio [62]. Laccase was also produced earlier when the fungus was cultivated in nitrogen rich media rather than nitrogen-limited media [63].

## 7. Induction of Laccase

Production of laccase can be considerably enhanced by addition of various supplements to the media [64]. The addition of xenobiotic compounds such as xylidine, lignin, and veratryl alcohol increased and induced laccase activity [65]. In one study by Lu et al. [66] it was observed that addition of cellobiose can induce appreciable laccase activity in some species of *Trametes*. Low concentration of copper was also shown to exhibit inducible effect on laccase activity [67]. Various basidiomycetes, ascomycetes, and deuteromycetes grown in sugar rich liquid medium were induced for laccase production by the addition of 2,5-xylidine. It was explicitly demonstrated that cultures of *Fomes annosus*, *Pholiota mutabilis*, *Pleurotus ostreatus*, and *Trametes versicolor* were stimulated for laccase production by addition of xylidine, and in the case of *Podospora anserina* rather decrease in activity was observed by xylidine addition [68].

## 8. Influence of pH and Temperature on Laccase Production

The information on effect of pH and temperature effects on laccase production is scarce, but most reports indicate initial pH between 4.5 and 6.0 that is suitable for enzyme production [6]. The optimum temperature for laccase production is between 25°C and 30°C [69]. When fungi were cultivated at temperatures higher than 30°C the activity of enzyme was reduced [70].

## 9. Type of Cultivation

Laccases have been produced vividly in both submerged and solid state modes of fermentation.  Table 1 lists the different cultivation techniques that have been adopted for large-scale production of laccase using wild-type filamentous fungi. In forthcoming sections, important features of laccase production into two modes, submerged and solid, state will be discussed.

## 10. Submerged Fermentation

Submerged fermentation involves the cultivation of microorganisms in liquid medium containing appropriate nutrients with high oxygen concentrations when operated in aerobic conditions. One of the major challenges in fungal submerged fermentations is viscosity of broth. Mycelium formation during growth of fungal cells can also impede impeller action causing blockades resulting in oxygen and mass transfer limitations. Different strategies have been employed to deal with oxygen and mass transfer limitations. A pulsed system developed by [71] to contain overcontrolled growth has been employed in decoloration of synthetic dye by the white rot fungus *Trametes versicolor *[72–75] allowing bioreactor to operate in continuous mode for prolonged times with high efficiency. Cell immobilization is another technique to alleviate problems associated with broth viscosity, and oxygen and mass transfer. Schliephake et al. [42] produced laccase by *Pycnoporus cinnabarinus *immobilized on cubes of nylon sponge in a 10-L packed bed bioreactor operated in a batch mode. Luke and Burton [44] reported that the immobilization of the fungus *Neurospora crassa *on membrane supports allowed the continuous production of laccase for the period of four months without enzyme deactivation. Sedarati et al. [76] compared the free cell cultures of *T. versicolor* with immobilized cultures using nylon mesh for the bioremediation of pentachlorophenol (PCP) and 2,4-dichlorophenol (2,4 DCP). Authors observed that immobilized cultures led to efficient removal. Couto et al. [77, 78] investigated different synthetic materials as carriers for the immobilization of the white rot fungus *Trametes hirsuta *in fixed bed bioreactors operated in batch. They found that among the different materials tested, stainless steel sponge led to the highest laccase activities. Park et al. [79] found that immobilization of the white rot fungus *Funalia trogii *in Na-alginate beads allowed the efficient decolouration of dye Acid Black 52. Other factors affecting the laccase production is agitation. Hess et al. [80] found that laccase production by *Trametes multicolor *decreased considerably when the fungus was grown in stirred tank reactor, presumably because of damage to mycelia. Mohorčič et al. [81] found that it was possible to cultivate the white rot fungus *Bjerkandera adusta *in a stirred tank reactor after its immobilization on a plastic net, although very low activities were attained. Tavares et al. [82] on contrary observed that agitation did not play an important role in laccase production by *T. versicolor*. Fed-batch mode of operation is also shown to be an effective way of producing laccase. Galhaup et al. [43] found that operating in fed batch increased the laccase production of *T. pubescens *by twofold and obtained a higher laccase activity.

## 11. Solid State Fermentation

Solid state fermentation (SSF) is defined as fermentation process occurring in absence or near absence of free liquid, employing an inert substrate (synthetic materials) or a natural substrate (organic materials) as a solid support [83]. SSF is shown to be particularly suitable for the production of enzymes by filamentous fungi because they mimic the conditions under which the fungi grow naturally [83, 84]. The use of natural solid substrates, especially lignocellulosic agricultural residues as growth substrates has been studied for various enzymes like cellulases [85, 86] including laccases [87]. The presence of lignin and cellulose/hemicellulose act as natural inducers and most of these residues are rich in sugar promoting better fungal growth and thus making the process more economical [8]. The major disadvantage with SSF is lack of any established bioreactor designs. There are several bioreactor designs that exist in the literature that have addressed the major limitations of heat and mass transfer in solid media. Nevertheless lot of progress is still to be made. Different bioreactor configurations have been studied for laccase production. Couto et al. [45] tested three bioreactor configurations immersion, expanded bed and tray for laccase production by *T. versicolor* using, and inert (nylon) and noninert support (barley bran). They found that the tray configuration led to the best laccase production. Couto et al. [46] also compared tray and immersion configurations for production of laccase by *T. hirsuta* using grape seeds as substrate. Tray configuration gave the best results here as well, and in a similar study by Rosales et al. [88] tray configuration produced higher laccase activity in *T. hirsuta* cultures raised on orange peels.

## 12. Applications of Laccase in Food Processing

Table 2 shows the multiple applications of laccases in the food industry. Areas of the food industry that benefit from processing with laccase enzymes include baking, juice processing, wine stabilization, and bioremediation of waste water [52]. The use of laccase enzymes allows for the improvement of functionality along with sensory properties. Laccase can also be utilized for analytical applications including biosensors, enzymatic, and immunochemical assays [51].

The baking industry utilizes a variety of enzymes to improve bread texture, volume, flavor, and freshness along with improving machinability of dough during processing. Bt the addition of laccase to dough used for baked products, the enzyme exhibits an oxidizing effect resulting in improved strength of gluten structures in dough and baked products. It has also been found that the addition of laccase results in increased volume, improved crumb structure, and softness of baked products. Machinability of dough was also found to be improved due to increased strength and stability along with reduced stickiness with the addition of laccase. Improved bread and dough qualities with the addition of laccase were also seen when used with low quality flours [89]. 

Due to the growing awareness of celiac disease (CD), increased interest has focused on the development of gluten-free baked products. CD is an immune-mediated enteropathy triggered by the ingestion of gluten, contained in many cereal flours including wheat, rye, and barley, by genetically susceptible individuals. Cereal flours, like oats and starches such as rice, potato, and corn, have been the focus for the development of gluten-free baked products [90]. These flours and starches lack the protein matrix responsible for dough formation and physical characteristics found in wheat-based baked products. Mimicking the protein matrix formed by the gluten proteins during dough formation of wheat flour has become exceedingly complex. Recent research has focused on using gluten-free oat flour along with enzymes to produce baked products acceptable for CD patients. The addition of laccase and proteolytic to oat flour lead to a significant improvement to texture quality of oat bread, due to increased loaf specific volume and lowering crumb hardness and chewiness. Chemical analysis of oat flour batter treated with laccase and proteolytic enzyme were found to cause a *β*-glucan depolymerisation and protein polymerization, resulting in improved rheological properties and positively contribute to improved bread making performance by oat flour [49].

Laccase is also commonly used to stabilize fruit juices. Many fruit juices contain naturally occurring phenolics and their oxidation products, which contribute to color and taste. The natural polymerization and cooxidation reactions of phenolics and polyphenols over time results in undesirable changes in color and aroma. The color change, referred to as enzymatic darkening, increases due to a higher concentration of polyphenols naturally present in fruit juices [91]. Research by Giovanelli and Ravasini [47] utilized laccase in combination with filtration in the stabilization of apple juice. Treatment with laccase caused the removal of phenols with high efficiency compared to other methods, like activated coals. The substrate-enzyme complex is then removed via membrane filtration, a critical treatment process. Color stability was found to be greatly increased after treatment with laccase and active filtration, although turbidity was present. The phenolic content of juices has been found to be greatly reduced after treatment with laccase along with an increase in color stability [91]. Laccase treatment has also been found to be more effective for color and flavor stability compared to conventional treatments, such as the addition of ascorbic acid and sulphites [89]. 

The high concentration of phenolics and polyphenols also come into play during wine production, particularly the crushing and pressing stages. The high concentration of polyphenols from stems, seeds, and skins contribute to color and astringency and are dependent on grape variety and vinification conditions [48]. The complex sequence of events resulting in the oxidation of polyphenols occurs in musts and wines causing flavor alterations and intensification of color in red wines. This phenomenon is also known as maderization [92]. Multiple methods can be utilized to prevent madeirization and they include catalytic factors, block oxidizers or the removal of polyphenols via proteinaceous, clarification, polyvinylpolypyrrolidone (PVPP) and high doses of sulfur dioxide [48]. However, research by Minussi et al. [48] found that treatment with laccase for the removal of polyphenols should be selective, as indiscriminate removal can result in undesirable organoleptic characteristics. Minussi et al. [48] further concluded that treatment of white wines with laccase is feasible and could diminish processing costs and increase storability of white wines over extended periods of time.

The use of laccase for stabilization is not limited to wine; the beer industry has potential to benefit from laccase treatment. Classic haze formation in beer is attributed protein precipitation stimulated by proanthocyanidins polyphenols, which are naturally present in small quantities [92]. This complex formed is commonly referred to as chill haze, which occurs upon cooling of the beer. The complex can be redissolved by warming of the beer to room temperature or above. However, after extended periods of time, protein sulphydryl groups replace phenolic rings and lead to permanent haze that does not redissolve at room temperature [89]. Traditionally, excess polyphenols are removed via PVPP treatment, however, PVPP is difficult to handle and creates problems in waste water treatment due to its low biodegradability. Laccase has been identified as easier to handle and safer for the oxidation of polyphenols in wort [89]. The addition of laccase at the end of processing has the added benefit of the removal of polyphenols and excess oxygen present; reduced oxygen content results in a longer shelf life of beer [92].

Since laccase are capable of degrading phenolic compounds, utilization for bioremediation of food industry wastewaters is vital. Bioremediation includes processes and actions used to biotransform an environment altered by contaminants back to its original status [93]. Many countries heavily regulate pollutants, including the class of aromatic compounds, which includes phenols and amines [50]. Research by Minussi et al. [48] reported the removal of naturally occurring and xenobiotic aromatic compounds from aqueous suspensions using immobilized laccase on organogel supports. The application laccase for bioremediation of wastewater streams is particularly of interest to beer factories. Fractions of wastewater released from beer factories contain a large amount of polyphenols and are dark brown in color. Research by Yagüe et al. [94] found laccase produced by the white rot fungus *Coriolopsis gallica* was capable of degrading polyphenols present in wastewater. Other research by González et al. [95] utilized laccase from *Trametes* sp. for the bioremediation of distillery wastewater generated from the ethanol production from the fermentation of sugarcane molasses with a high content of organic matter and an intense dark-brown color. Bioremediation of olive mill wastewaters via immobilized laccase has also been reported. It has also been found that utilizing olive oil mill wastewaters has been beneficial in the cultivation of fungi for laccase production [89].

## 13. Conclusions

Laccases are versatile oxidases, and their versatility lies in the high reduction potential that makes them potential candidate for biotechnological applications, especially for the food industry. Laccases have the potential to make food processing more economical and environmental friendly. To proficiently realize this potential it would require more efficient laccase production systems and better understanding of their mode of action. With the use of mediators it is possible to extend the role of laccase to nonphenolic substrates. Extensive occurrence of laccase in various fungal genera ensures their widespread availability, and especially the wood rotting basidiomycetes also referred as white rot fungi are the excellent laccase producers. Overexpression of laccases in heterologous systems has been actively pursued to enhance their titers and to improve their catalytic activity. Media optimization and use of appropriate inducers could bring additional benefits of higher production with expenditure of minimum resources. Both submerged and solid state cultivation techniques have been embraced by the researchers for laccase production. Submerged fermentation, though, leads the SSF for industrial production of laccase. Future efforts in improving the SSF bioreactor designs can make SSF more potent and competitive. With plethora of applications in food processing including baking and role in gluten-free breads, beverage (wine, juice and beer) stabilization, and bioremediation, laccases certainly have important role to play in green food processing.

## Figures and Tables

**Table 1:** Production of laccases in different cultivation modes.

Fungi	Type of cultivation	Inducer	Laccase Activity (U/L)	Reference
*Pycnoporus cinnabarinus*	Submerged	10 mM Veratryl alcohol (VA)	280	[42]
*Trametes pubescens*	Submerged	2 mM Cu^2+^	333,000	[43]
*Neurospora crassa*	Submerged	1 *μ*M cyclohexamide	10,000	[44]
*T. versicolor*	SSF (Immersion, nylon sponge)	Tween 80	229	[45]
*T. versicolor*	SSF (Immersion, barley bran)	Tween 80	600	[45]
*T. versicolor*	SSF (Expanded bed, nylon sponge)	Tween 80	126	[45]
*T. versicolor*	SSF (Expanded bed, barley bran	Tween 80	600	[45]
*T. versicolor*	SSF (Tray, nylon sponge)	Tween 80	343	[45]
*T. versicolor*	SSF (Tray, barley bran)	Tween 80	3500	[45]
*T. hirsuta*	SSF (Tray, grape seeds)	—	18,715	[46]

**Table 2:** Laccase producing organism and biotechnological application for use.

Laccase Producing Organism	Application	Reference
*Trametes versicolor*	Filtration aid	[47]
*Trametes versicolor*	Wine stabilization	[48]
*Myceliophthora thermophilia*	Dough conditioner	[49]
*Rhizoctonia praticola*	Phenolic compound removal	[50]
*Trametes versicolor; Rhizoctonia praticola*	Soil decontamination	[50]
*Coriolopsis gallica*	Beer factory waste water	[51]
*Trametes sp.*	Distillery waste water	[51]
*Trametes versicolor; Pleurotus ostreatus*	Olive Mill wastewaters	[51]
*Trametes hirsuta*	Dough Conditioner	[52]
